# Prevention vs treatment of rheumatoid arthritis

**DOI:** 10.1093/immadv/ltad016

**Published:** 2023-08-29

**Authors:** Lars Klareskog, Lars Alfredsson

**Affiliations:** Rheumatology Division, Department of Medicine, Solna, Karolinska Institutet, Stockholm, Sweden; Institute of Environmental Medicine, Karolinska Institutet, Stockholm, Sweden

**Keywords:** Prevention, Rheumatoid arthritis, Autoimmunity, Tolerance, Immunotherapy

## Abstract

Whether a yet chronic and not curable disease like rheumatoid arthritis (RA) can be subject to prevention or whether available resources should be focused on treatment is a classical dilemma. Similar to the case in most other chronic diseases, the focus in research as well as in clinical practice has been on the treatment of established diseases, resulting in drugs that are efficient in eliminating most joint damage but not able to cure the disease or stop needs for continuous treatment of the disease. Less effort has been spent on identifying and implementing ways to prevent the disease. We argue in this review that knowledge concerning the longitudinal evolvement of the major, ‘seropositive’ subset of RA has now come to a stage where prevention should be a large part of the research agenda and that we should prepare for prevention as part of clinical practice in RA. We describe briefly the knowledge basis for broad public health-based prevention as well as for a ‘precision prevention’ strategy. In the latter, individuals at high risk for RA will be identified, monitored, and ultimately provided with advice on how to change lifestyle/environment or be given treatment with drugs able to delay and ultimately stop the development of RA. Whether this potential of precision prevention for RA will change the broader clinical practice will depend on whether specific and long-lasting interference with disease-inducing immunity, ultimately ‘tolerance therapy’, will become a reality.

## Introduction

Prevention of a disease is by definition always better than treatment when the disease has already occurred and caused subjective symptoms for patients and demands for resources from health care. Prevention can be divided into two broad categories: The first is broad public health-based prevention where knowledge about causative and modifiable factors in environment and/or lifestyle are known; a prototype is smoking and lung cancer. The second is prevention directed towards individuals at high risk for a particular disease where causative mechanisms leading from the risk to the disease state are known and where also interventions aimed at prevention are known to be effective; prototypes are the exclusion of gluten from the diet in coeliac disease and anti-hypertensive treatment for prevention of ischemic heart disease. We sometimes name such focused prevention efforts ‘precision prevention’.

Immune-mediated diseases like RA have so far not been major targets for such prevention efforts neither at the public health nor at the precision prevention level. In this short review, we will argue that both roads to prevention should be worked on and introduced but also that specific problems are associated with both approaches. For public health-based prevention, we need to balance costs and acceptance of the potential action for prevention. For precision prevention, we need to balance the benefit of prevention of disease in some individuals against the harm preventive measures would cause in others who might never get disease even without treatment. Below follows some considerations on prevention of RA taking both public health-based and precision prevention into account.

## Public health-based prevention of RA; potentials and obstacles

Prerequisites for public health-based prevention are on one hand solid data on modifiable risk and protective factors in environment and/or life styles and on the other structures and attitudes in society that enable prevention based on these data to take place in reality. Again, smoking and lung cancer provides an illustrative example where initial and solid data on the connection preceded the societal implementation of broad information campaigns and smoke-less environment for many years [1].

For RA, it is known since the 1990s that cigarette smoking comprises a risk for RA [2], but the insight was not widely disseminated. The extent of the impact of smoking on risk for RA and that the risk could be very high in individuals with certain genotypes was demonstrated only a decade later. Here, it was shown that cigarette smoking in combination with certain MHC (Major Histocompatibility Complex) class II variants (named ‘shared epitope, SE’) increased the risk for seropositive (with rheumatoid factors [RF] and/or antibodies to citrullinated protein antigens [ACPA]) with up to 40 times in heavy smokers [3–7]. Further, the so-called ‘attributable proportion of RA due to smoking’ was found to be around 20% for all RA, 35% for ACPA-positive RA, and 55% for individuals with a risk genotype [8]. Thus, with the smoking habits at the time, more than half of ACPA-positive RA would never have happened in the genetically RA-pre-disposed individuals if nobody had smoked. Important for the determination of causality was the information that cessation of smoking leads to a dose-dependent diminished risk for RA, even if this takes several years [9, 10]. This example illustrates one of the points in this review, namely that epidemiologic studies on modifiable risk factors for RA – as well as for many other immune-mediated diseases – have historically not been prioritized very high in the research community. Thus, much might be gained from better knowledge on modifiable risk factors for RA and on how such knowledge can be used for the prevention of the disease. So, what is the current status of environmental, preferably modifiable risk and protective factors for RA?

A summary of currently known airway risk factors is provided in Table 1. Except for tobacco smoke, the most notable and best-studied risk factors are noxious inhalable substances that occur within as well as outside occupational life. Such common risk factors include gasoline and diesel exhausts [11], silica [11–19], textile dust [20, 21], and other types of dust present in some occupations [11, 16, 18, 22–25] and herbicides and other dusts common in agriculture [11, 26–28]. What was striking from one of the recent publications on these factors was both how these common exposures alone cause significant risk increase for seropositive RA and how this risk is much increased when combined with cigarette smoking and further amplified in individuals with certain common genetic features [11]. Notable, however, data on the most obvious and common inhalable potential risk factors, i.e. air pollution are partly conflicting [29–34] and have not yet been systematically investigated in large enough epidemiological studies.

**Table 1.** Airborne environmental factors associated with risk of developing RA

Exposure	RA overall	Seropos RA	Seroneg RA	References
Smoking	+	+	+/−	[2–10]
Occup exp to diesel exhaust	+	+	?	[11]
Occup exp to gasoline exhaust	+	+	+	[11]
Occupational exp to silica	+	+	+	[11–19]
Occup exp to textile dust	+	+	+	[20, 21]
Occup exp to asbestos	+	+	+	[11, 16, 18, 22, 23]
Occup exp to coal	+/−	?	?	[24, 25]
Occup exp to herbicides	+	?	?	[11, 26–28]
Air pollution (traffic)	+/−	?	?	[29–34]

Effects are shown for RA without any subsetting, and for the ‘seropositive’ and ‘seronegative’ subsets. ‘seropositive is in this context patients positive for ACPA and/or RF; for many exposures differentiation between RF-positive and ACPA-positive groups has not been possible. In the table, (+) means that exposure for the environmental factor(s) has been shown to increase risk for RA, whereas (−) means that no risk has been observed. For some exposures, data are conflicting with different results from different studies and such exposures are denoted (+/−). In cases no well-documented studies exist and such exposures for RA or RA subset are assigned with (?). The table is based on a systematic review and a thorough investigation of published articles within the area using the PubMed search system.

There are several additional environmental/lifestyle-based risk factors for RA described although not with the same magnitude of risk as for the airway exposures. Thus, the second most investigated risk factor for RA is periodontal disease, and bacteria in gums associated with periodontitis [35–37]. Here, both *Porphyromas gingivalis* and other bacteria have been implied as risk factors for RA based on both epidemiology and cross-reactivity between such bacteria and citrullinated self-antigens [35–37]. It is not yet clear whether and to which extent effective measures against peridontitis will reduce the risk for RA, even if some studies point in this direction [38]. Finally, there are a number of potential risk factors that have not been systematically confirmed in enough big independent studies; such potential risk factors include certain meat-containing diets [39] and working in cold and wet environments [40].

As an obvious addition to risk factors, also protective modifiable factors need to be investigated, and also here there is a relative lack of big and conclusive studies. The most solid data exist for alcohol consumption where several cohort and case–control studies have shown a dose-dependent reduction of risk for RA associated with alcohol use [41, 42]. Also, certain diets, in particular fish and omega-3-containing diets are associated with some protection against RA [43]. Of particular interest here is whether the effects of these protective food items can be explained by mechanisms that can be used for life-style-based or drug-based prevention; the demonstration of the pronounced effects of alcohol and its metabolites on both adaptive and innate immunity may help us to identify such targetable molecular pathways [42]. However, available data do not yet provide enough evidence for broad dissemination of information on most of the so far described protective and risk environments/lifestyles except for those involving noxious effects on airways.

A general conclusion from these observations is that any focus on the prevention of RA should include requests for more epidemiological studies on risk factors for the disease, coupled with the establishment of structures that disseminate results from such studies to society. First, studies on risk and protective factors for a disease requires substantial investments in big epidemiological studies, i.e. in both cohort and case-control studies. So far, investments in these areas from research funding agencies are limited. Second, also communication systems for dissemination of correct, useful, and actionable preventive measures are needed, but today rarely directed towards immune-mediated diseases. We mean that priorities in both these areas need to be shifted.

## Precision prevention-based approaches

### RA is a disease where much is known about initial triggering of disease-associated immunity

In an immunological perspective, the most interesting and challenging feature of RA is that immune reactions, measured as the presence of RF and ACPA precede the development of seropositive RA, and that much knowledge has been gained concerning how these immune reactions are triggered and perpetuated (for reviews, see [44, 45]. Thus, at least two complementary mechanisms behind the triggering of immunity to citrullinated as well as other post-translationally modified antigens (by carbamylation and acetylation) have been described. First, noxious substances such as cigarettes smoke and silica dust can induce increased expression and function of enzymes, peptidyl-arginine-deiminases, followed by citrullination of many proteins in the lung or oral mucosa [46, 47]. Second, bacteria, not only *Porphyromonas gingivalis*, but also other species can express citrullinated or other post-translationally modified molecules followed by triggering of immunity with antibodies that may cross-react between bacteria and self-antigens [38, 48]. Both noxious substances and bacteria can contribute to the activation of antigen-presenting cells, which in individuals with certain MHC class II allotypes can lead to activation of T- and B-cell responses against these post-translationally modified antigens [49, 50]). Evidence that T-cell-dependent B cell activation indeed occurs in the lungs comes also from the demonstration that highly somatically mutated ACPAs have been cloned from lungs of patients with RA as well as from ACPA-positive individuals at risk for RA [51]. Other experiments have demonstrated a sustained activation of B cells producing antibodies against citrullinated and other post-translationally modified antigens during long times before the onset of arthritis [52]. These emerging insights into mechanisms behind RA-associated autoimmunity should provide opportunities to intervene with such mechanisms.

### Symptoms that may occur before arthritis in ACPA-positive individuals

It was early shown that ACPA-positive individuals without arthritis often show signs of pain, in particular pain in joints – arthralgia – and that presence of this symptom increased the risk for RA in ACPA-positive individuals [53]. The subsequent demonstration that ACPA, both polyclonal IgG from patients and monoclonal IgG from patient-derived B cells and plasma cells, could induce pain behavior in mice [54–56], indicated a mechanistic link between these two features, strengthening the concept of using both arthralgia and presence of ACPA for prediction of risk for RA. The degree of predictability of future disease – here RA – is a fundamental part of the judgement of the risk-benefit ration for prevention therapies, and thus much effort has been made to improve predictability for risk for RA. Thus, certain features of the ACPAs, such as the presence of glycosylation of Fab and Fc parts of ACPA [57, 58] are predictive for high risk of future RA, as is also emerging signs of bone loss and subtle signs of inflammation [59]. Nevertheless, we do not yet have any definite combination of symptoms and biomarkers that predict the onset of RA. Instead, recent experimental data have shown that some ACPAs, both monoclonal and polyclonal are able to prevent rather than, as previously anticipated, promote the development of arthritis – so far in a murine experimental model of arthritis [60, 61]. More precisely, it was shown that some monoclonal and murinized ACPAs, derived from cloning of Ig genes from single B cells/plasma cells from RA and at risk for RA patients, prevented from arthritis induced by anti-collagen II antibodies, whereas other monoclonals with other fine specificities enhanced arthritis development [60]. These data indicate that at least the humoral immune response occurring before the onset of RA may have more diverse functions than previously recognized. Thus, additional studies on the potential generealisability of these findings to other mouse models and to the human situation may both lead to better diagnostics for the prediction of who are at the highest risk for RA, and to knowledge on what immune interventions may change the balance between disease-inducing and disease-protective immunity.

### Balancing benefit vs risk in precision prevention for RA

As described above, RA offers a very interesting case for precision prevention based on the presence of antibodies and symptoms before the onset of arthritis and based on emerging knowledge on where and how disease-associated immunity can be triggered and propagated. Nevertheless, the balance between the potential harm and the potential gains has to be considered in any intervention. So far, observational studies on the effects of lifestyle changes in individuals at high risk for RA using knowledge of modifiable environmental/lifestyle factors indicate the interventions with lifestyle changes may have substantial preventive effects [62]. Gains are obvious, but risks are also present as in all new interventions in healthcare, in particular for individuals who might, after all, never develop disease. One such risk is that individuals identified as having an increased risk for RA will have increased worries and identify her/himself as a ‘diseased’ individuals feel RA. Such worries must be taken seriously, and several research programs have addressed this issue and suggested action to be taken in carefully designed information programs and emphasis on healthy lifestyles. Another potential drawbacks of screening programs to identify individuals at risk for RA are the allocation of health care resources to programs of as yet uncertain long-term benefits at the expense of care of established disease. Such problems are best handled if prevention programs can be driven with the help of digital tools and major patient participation, thereby minimizing the need for health care resources needed elsewhere. Such programs aimed at digital care for prevention are presently ongoing and supported by the EU grant system EITHealth. Notably, the effects and adverse effects of such programs need to be evaluated as for any other type of intervention aimed to improve health.

Interventions with drugs aimed at intervening with disease-inducing immunity provide more challenges, where the degree of precision in predicting risk for disease onset is one factor, potential harms of treatment another. One very informative study in this respect was published recently, where a combination of methotrexate and corticosteroids was able to delay the onset of arthritis, but equally importantly showed the ability to reduce symptoms, i.e. pain and fatigue in individuals recruited to the study based on presence of these symptoms [63]. These data illustrate that knowledge of symptoms appearing before the onset of arthritis can be used both for prediction of risk for arthritis and as a symptom in need of therapy. Several studies are ongoing using immunotherapies for the prevention of RA, and if they point in the same direction as the previously published study using anti-CD20 antibody therapy, which delayed but did not inhibit arthritis development [64], there will be improved proof of concept that immunotherapy may be used to interfere with RA development. But currently available results, i.e. lack of long-term effects also point to the need to develop even more specific therapies aimed at more permanently changing arthritis-inducing immunity.

## Prevention vs therapy

A pertinent question that remains after this overview of possible ways to prevent RA is whether any of these potentials can offer cost-effective and feasible ways to address unmet needs in RA or whether we still should focus on therapy while prevention has to wait for better ways to re-regulate or eliminate disease-inducing immunity. Obviously, this is a question that has to be addressed experimentally with clinical trials linked to health economic studies. However, there is an additional argument for establishment of infrastructures for prevention that include identification of individuals at risk for RA and enabling individuals at risk for RA to monitor their symptoms and evolution of risk. Thus, a large current problem for effective treatment of RA is that it may take several months between the first subjective symptoms noted by patients, until proper diagnosis and treatment by a rheumatologist. Such delays, even with weeks or a few months, may negatively affect the disease course over several years to come [65].

We argue that an infrastructure established for the identification and monitoring of individuals at risk for RA would be very useful in enabling very early therapy for those who actually develop RA. Individuals identified as being at high risk for RA will, in this scenario, be provided with information on how to detect the first signs of arthritis and also get access to procedures for rapid contact with rheumatology care upon the first signs of arthritis. Seen in this way, identification and surveillance programs for individuals at risk for RA will not only enable prevention of disease for some but will also enable those not prevented from RA to get very early access to effective RA treatment, and thus to better long-term prognosis.

## Concluding remarks

**Figure 1.**
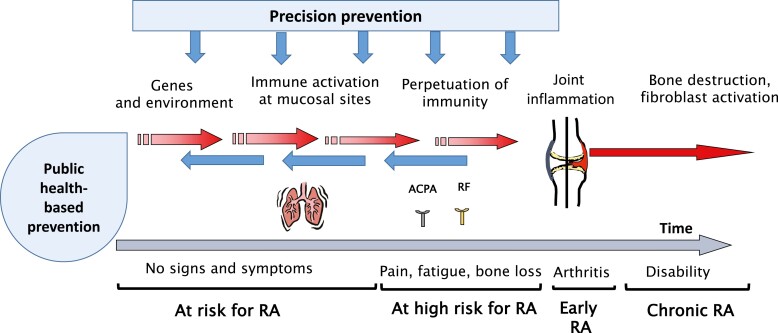
The figure illustrates the two major ways for prevention of RA: (1) Public health based prevention based on knowledge of environmental and life style factors that confer increased risk vs protection of RA; (2) Precision prevention based on knowledge of immunity that may promote vs counteract development of RA as well knowledge on environment and lifestyle involved in the different phases of development vs non-development of RA..

We argue in this short review that prevention of RA is becoming a feasible and meaningful aim for rheumatologists and that at least some objections towards trials for prevention can be met with arguments highlighted in this article, see also Figure 1. We also argue that it is now highly meaningful to identify and monitor individuals at risk for RA, both in order to support the development of future preventive therapies and in order to enable more efficient and earlier use of existing therapies for those who develop RA despite efforts for prevention. Notably, all such new interventions need to be carefully evaluated both for the effects and adverse effects of the identification process *per se* and for the potential interventions.

## Data Availability

Data from our laboratory that are referred to are available from the authors in case not already published in referenced papers. All content can be reproduced, provided permissions from the publisher.

## Abbreviations:

ACPA: Antibodies to citrullinated protein antigen
RA: Rheumatoid arthritis
RF: Rheumatoid factor
